# How large is the lung recruitability in early acute respiratory distress syndrome: a prospective case series of patients monitored by computed tomography

**DOI:** 10.1186/cc10602

**Published:** 2012-01-08

**Authors:** Gustavo FJ de Matos, Fabiana Stanzani, Rogerio H Passos, Mauricio F Fontana, Renata Albaladejo, Raquel E Caserta, Durval CB Santos, João Batista Borges, Marcelo BP Amato, Carmen SV Barbas

**Affiliations:** 1 Adult ICU Hospital Israelita Albert Einstein, São Paulo, 05652-000, Brazil; 2 Radiology Department, Hospital Israelita Albert Einstein, São Paulo, 05652-000, Brazil; 3 Respiratory ICU, Hospital das Clínicas and LIM-09, University of São Paulo, Brazil; 4 Department of Surgical Sciences, Section of Anaesthesiology & Critical Care, Uppsala University, SE-751 85, Uppsala, Sweden

## Abstract

**Introduction:**

The benefits of higher positive end expiratory pressure (PEEP) in patients with acute respiratory distress syndrome (ARDS) have been modest, but few studies have fully tested the "open-lung hypothesis". This hypothesis states that most of the collapsed lung tissue observed in ARDS can be reversed at an acceptable clinical cost, potentially resulting in better lung protection, but requiring more intensive maneuvers. The short-/middle-term efficacy of a maximum recruitment strategy (MRS) was recently described in a small physiological study. The present study extends those results, describing a case-series of non-selected patients with early, severe ARDS submitted to MRS and followed until hospital discharge or death.

**Methods:**

MRS guided by thoracic computed tomography (CT) included two parts: a recruitment phase to calculate opening pressures (incremental steps under pressure-controlled ventilation up to maximum inspiratory pressures of 60 cmH_2_O, at constant driving-pressures of 15 cmH_2_O); and a PEEP titration phase (decremental PEEP steps from 25 to 10 cmH_2_O) used to estimate the minimum PEEP to keep lungs open. During all steps, we calculated the size of the non-aerated (-100 to +100 HU) compartment and the *recruitability *of the lungs (the percent mass of collapsed tissue re-aerated from baseline to maximum PEEP).

**Results:**

A total of 51 severe ARDS patients, with a mean age of 50.7 years (84% primary ARDS) was studied. The opening plateau-pressure was 59.6 (± 5.9 cmH_2_O), and the mean PEEP titrated after MRS was 24.6 (± 2.9 cmH_2_O). Mean PaO_2_/FiO_2 _ratio increased from 125 (± 43) to 300 (± 103; *P *< 0.0001) after MRS and was sustained above 300 throughout seven days. Non-aerated parenchyma decreased significantly from 53.6% (interquartile range (IQR): 42.5 to 62.4) to 12.7% (IQR: 4.9 to 24.2) (*P *< 0.0001) after MRS. The potentially recruitable lung was estimated at 45% (IQR: 25 to 53). We did not observe major barotrauma or significant clinical complications associated with the maneuver.

**Conclusions:**

MRS could efficiently reverse hypoxemia and most of the collapsed lung tissue during the course of ARDS, compatible with a high lung recruitability in non-selected patients with early, severe ARDS. This strategy should be tested in a prospective randomized clinical trial.

## Introduction

Severe ARDS still has a very high mortality rate despite all advances in critical care [1]. Ventilator Induced Lung Injury (VILI) plays a major role in the poor prognosis of such patients, as demonstrated by extensive experimental and clinical evidence [2-10]. But there is no consensus yet on the least harmful mechanical ventilation to be individually applied at bedside [11-18]. Although most studies indicate that reducing inspiratory pressures and tidal volumes is generally beneficial, many uncertainties remain about the actual impact of the PEEP selection. Abundant physiological data suggest that the combination of recruitment maneuvers and individual PEEP titration is essential to optimize the effects of PEEP, but the proof of such benefits in humans is not yet conclusive [11,12,19-22].

Recently, three large clinical trials [13-15], including Acute Lung injury/ARDS patients ventilated with low tidal-volume, have compared different PEEP strategies (high vs. low), but none of them could show a significant difference in mortality. A recent meta-analysis has pooled those trials [23], revealing some combined benefit of the high PEEP strategy; still, the survival benefit was modest and limited to the subgroup of ARDS patients. Conceptually, one could argue that none of the "high-PEEP" strategies was designed to test the "open-lung hypothesis" postulated by Lachmann [24-27], that is, the hypothesis that most of the collapsed lung tissue observed in early ARDS can be reversed at an acceptable clinical cost, potentially resulting in better lung protection. According to a recent study by Borges and colleagues [22,28], a straight test of the "open-lung hypothesis" would certainly require more aggressive recruiting maneuvers in association with individualized, decremental PEEP titration. Thus, one can speculate that the limited results reported above were related to sub-optimal ventilatory strategy.

Although the study by Borges and colleagues [22] provided a good description of the physiological principles of the Maximum Recruitment Strategy (MRS), there is still a lack of information regarding the long term management, side effects, and generality of such findings in a non-selected population. In fact, a recent study by Gattinoni and colleagues [29] with 68 ALI/ARDS patients submitted to recruitment strategies, all under close monitoring by computed tomography (CT), has cast doubts about the feasibility of the "open-lung" strategy, since the recruitability of the lungs reported in this study varied too much among patients, amounting to less than 15% in most patients.

Thus, the objective of this study was to describe the feasibility and long term impact of the MRS applied in a case-series of non-selected patients with severe ARDS. All patients were closely monitored by multi-slice CT during the implemented strategy. Since the maneuver was individualized and applied in a more intensive fashion than in previous studies, we tried also to provide a more precise, bedside estimation of lung recruitability in a general population of patients with early ARDS.

## Materials and methods

### Patients

This study was conducted in a general medical/surgical ICU with 42 beds at the Albert Einstein Hospital, São Paulo, Brazil. From January 2003 to June 2009, 69 consecutive patients were screened for early and severe ARDS, according to the inclusion criteria depicted below. The 51 patients who did not meet any exclusion criteria were studied. The hospital ethical committee granted approval to this study and informed consent was obtained from patients' relatives.

### Early and severe ARDS inclusion criteria

In addition to the ARDS definition of the American-European Consensus Conference 1994, three additional inclusion criteria were required: a) less than 72 hours onset; b) age between 14 and 80 years, and c) PaO_2_/FIO_2 _< 200, obtained with PEEP ≥ 10 cmH_2_O, FIO_2 _of 1.0 and pressure-controlled ventilation with driving pressure set at 15 cmH_2_O.

### Exclusion criteria

Exclusion criteria for this study includes cardiac arrest in the last 48 hours; persistent hemodynamic instability (defined as: mean arterial pressure < 70 mmHg, central venous saturation (ScvO_2 _< 70%), despite adequate pre-load optimization with intravenous fluids and vasopressors); formal contraindication of hypercapnia (for example, acute coronary disease, cardiac arrhythmias, intracranial hypertension); active bronchopleural fistula; incapacity to perform CT scan due to excess body weight (> 180 kg) or size (abdominal circumference > 200 cm); do not resuscitate orders; pregnancy; and denial from the family or assistant physician.

### Baseline mechanical ventilation settings

Baseline arterial blood gas was drawn with a minimum PEEP of 10 cmH_2_O (enough to keep SpO_2 _> 90%), pressure-controlled ventilation, driving-pressure of 15 cmH_2_O, respiratory-rate of 15 to 20 breaths/minute, inspired oxygen-fraction (FIO_2_) = 1.0.

### Transportation to CT facility

Patients were deeply sedated (midazolam and fentanyl) and paralyzed (cisatracurium). All patients were monitored with central venous (Arrow CV-17702-E-USA) and arterial (Arrow RA-04220-W-USA) lines, and submitted to a preload optimization protocol targeting pulse-pressure variations less than 13% [30-32] and ScvO_2 _greater than 70%. Patients were only transported after stabilization with fixed doses of vasopressors. A Servo 900c ventilator (Maquet, Solna, Sweden), equipped with a long term external battery, was used during transportation and CT scanning.

### Maximum Recruitment Strategy (MRS)

**Figure 1:**
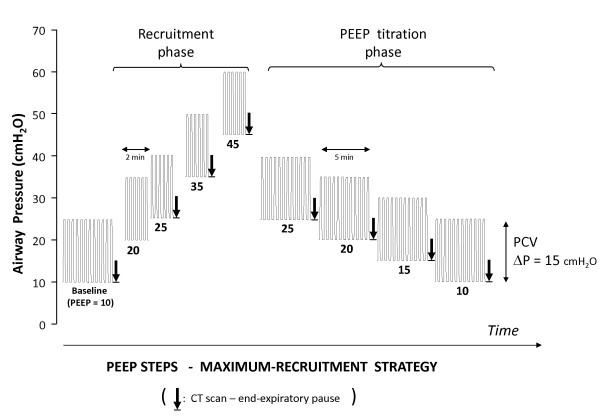
**Design of Maximum Recruitment Strategy (MRS) protocol**. The first phase, "recruitment phase", starts at baseline-PEEP (around 10 cmH_2_O) and goes up to maximum-PEEP (around 45 cmH_2_O), transiently generating plateau-inspiratory pressures around 60 cmH_2_O. The second phase, "PEEP titration phase", starts at PEEP = 25 cmH_2_O, and goes down to PEEP = 10 cmH_2_O. PCV: pressure controlled ventilation; ΔP: driving pressure = plateau inspiratory pressure minus PEEP. The arrows mark the timing of CT scanning during an expiratory pause at the designated PEEP.

All patients underwent MRS guided by thoracic CT scan as depicted in Figure 1. MRS consisted of two-minute steps of tidal ventilation with pressure-controlled ventilation, fixed driving-pressure = 15 cmH_2_O, a respiratory-rate of 10 to 15 breaths/minute, an inspiratory:expiratory ratio of 1:1 and stepwise increments in PEEP levels from 10 to 45 cmH_2_O (recruitment-phase). Then, PEEP was decreased from 25 to 10 cmH_2_O (PEEP titration-phase), in steps of 5 cmH_2_O, each one lasting four minutes.

At each of the steps marked in Figure 1, CT image sequences (Mx Twin e Mx 8000- Philips Medical Systems - Eindhoven, Netherlands) from the carina to the diaphragm were acquired during an expiratory pause of 6 to 10 seconds. The approximate position of the carina was identified in each step. Lung collapse was assessed on-line (by visual inspection, for immediate clinical decision) and off-line (for quantitative measurements described below).

For on-line visual assessment of collapse, a mediastinal window with a width of 400 HU and center at +50 HU was used. P_OPENING _was defined as the minimum inspiratory pressure needed to achieve negligible collapse, or the minimum amount of non-aerated tissue at visual inspection of thoracic CT at the most dependent lung regions during the recruitment phase. Afterwards, during the PEEP titration phase, P_CLOSE _was defined as the PEEP in which visual collapse started to recur. The recruitment phase could be aborted and immediately followed by the PEEP titration phase, in case of P_OPENING _< 60 cmH_2_O.

MRS was planned to be interrupted in the following situations: sustained drop, or a 30-second desaturation at arterial oxygenation (SpO_2_) < 90% after checking the adequacy of oximeter probe and finger perfusion, mean arterial pressure (MAP) < 65 mmHg, evidence of barotrauma (in CT images), or cardiac arrhythmias associated with cardiovascular collapse.

Patients were transported back to the ICU where they were submitted to a second MRS at ICU arrival, right after the ventilator change. After that, we performed no additional recruitment maneuvers, except if tidal volume decreased more than 20% at the same delta inspiratory pressure-control (considered as indicative of lung de-recruitment), or in case of ventilator disconnections. The team was explicitly instructed to avoid disconnections and depressurization. Closed systems for tracheal aspiration were always used and we tried to aspirate the patients as little as possible. After ICU arrival, patients were ventilated in pressure-controlled mode, at a PEEP level above P_CLOSE _(or 25 cmH_2_O - the lower of both - henceforth called titrated-PEEP), inspiratory-driving pressures ≤ 15 cmH_2_O whenever possible and the lowest possible FIO_2 _to keep SpO_2 _≥ 95%. Titrated-PEEP was maintained for at least 48 hours. Afterwards, PEEP was decreased in steps of 2 cmH_2_O every 8 to 12 hours, whenever PaO_2_/FIO_2 _was higher than 300. If PaO_2_/FIO_2 _decreased to less than 300 during attempts of PEEP reduction, a new recruitment maneuver was repeated, and PEEP was restored to its previous level.

After 48 hours, and provided that PEEP could be set below 20 cmH_2_O, sedation was reduced and pressure support ventilation was started. When both, pressure-support and PEEP levels could be reduced to 10 cmH_2_0, patients were extubated to non-invasive ventilation. When prolonged mechanical ventilation was anticipated, tracheostomy was performed. All patients were followed until death or discharge. Organ failure and sepsis definitions were the same as used by Villar and colleagues [12].

### Quantitative CT-image analysis

Quantitative CT analysis was performed off-line, as previously described [22]. Three situations were analyzed: baseline PEEP, maximum-PEEP (during P_OPENING _detection), and titrated-PEEP. The right and left lung regions were manually segmented, conservatively avoiding partial volume artifacts from heart, chest wall and great vessels. For each region of interest (ROI) (right and left lungs) we computed the number of voxels, with its respective mass and volume within and analyzed specifically non-aerated (-100 to +100 HU) density compartment.

### Lung recruitability analysis

The Potentially Recruitable Lung calculation was adapted from reference [29]. Attempting to minimize radiation dose, instead of whole lung analysis, we limited CT scanning to a significant lung fraction, a thick cross-section from the carina to the diaphragm, typically encompassing 7 to 8 cm of the craniocaudal axis (about 8 to 10 contiguous slices), and representing the largest cross-sectional area of the lung. Accordingly, the potentially recruitable lung was calculated as a fractional mass of lung tissue within this relevant section: the mass of collapsed tissue that could be re-aerated from baseline to maximum-PEEP, divided by the total lung mass within the section.

Relative-response to the maneuver was calculated as another fractional mass, using the same numerator as above, but divided by the mass of non-aerated tissue within the section, observed at baseline-PEEP.

### Statistical analysis

Quantitative variables were either presented as mean ± standard deviation (SD), or as median with interquartile range (for non-normal distributions). ANOVA for repeated measures (after appropriate log transformations) was used to compare the amount of collapse in different phases of the protocol. The Mann-Whitney test and Multivariate Logistic regression analysis were used to check the association between clinical, CT variables and outcome. Data were analyzed with the SPSS 13.0 version (IBM^®^, Armonk, New York, USA). The significance level considered was *P *< 0.05.

## Results

Demographic and clinical characteristics of the study population are shown in Table 1. Gas exchange and mechanical ventilation data are shown in Table 2 and hemodynamic data in Table 3

**Table 1:** Baseline patient characteristics (N = 51)

Age (years)	50.7 ± 16.5
**Sex (male)**	**65%**
**Primary/Secondary ARDS**	**84%/16%**
**Pneumonia** **Aspiration gastric contents**	**29 (57%)** **9 (17%)**
**Sepsis**	**5 (10%)**
**Thoracic trauma**	**2 (4%)**
**TRALI**	**2 (4%)**
**Other**	**4 (8%)**
**APACHE II score**	**20.2 ± 6.2**
**SOFA score (day 1)**	**10 (7 to 12)**
**NPOF**	**2 (1 to 2)**
**Sepsis**	**71%**
**Septic shock**	**63%**
**Vasopressor**	**82.3%**

**CRRT**	**56.8%**

Primary/Secondary ARDS, Primary ARDS/Secondary ARDS; TRALI, transfusion related Acute Lung Injury; NPOF, non-pulmonary organ failure; Vasopressor, percentage of patients receiving vasopressors at entry; CRRT, continuous renal replacement therapy. Data expressed as percentage (%), mean ± standard deviation, or median with interquartile range in brackets

**Table 2:** Gas exchange and mechanical ventilation data before and throughout the first week after MRS

	Before MRS	After MRS	Day 3	Day 5	Day 7
**PaO_2_/FiO_2_**	125 ± 43	302 ± 102	339 ± 142	345 ± 110	350 ± 102
**PaCO_2 _(mmHg)**	48 ± 13	56 ± 16	52 ± 13	44 ± 10	42 ± 9
**pH**	7.26 ± 0.11	7.20 ± 0.12	7.26 ± 0.10	7.37 ± 0.09	7.41 ± 0.07
**BE**	-5.7 ± 6.0	-7.2 ± 5.4	-4.7 ± 5.2	-0.5 ± 5.1	1.7 ± 4.1
**PEEP(cmH_2_O)**	11.7 ± 3.8	24.6 ± 2.9	23.1 ± 3.3	19.5 ± 4.3	17.2 ± 3.7
**Pplat (cmH_2_O)**	26.7 ± 3.8	39.9 ± 4.3	37.2 ± 4.4	32.8 ± 6.8	29.7 ± 6.3
**ΔP (cmH_2_O)**	15 ± 0	15.4 ± 3.4	14.1 ± 3.3	13.3 ± 4.6	12.3 ± 4.6
**V_T _(ml/kg)**	-------	6.9 ± 1.4	7.2 ± 1.9	8.6 ± 1.8	8.8 ± 1.5
**FiO_2 _(%)**	100	45 ± 17	41 ± 18	35 ± 11	37 ± 16

ΔP, driving pressure; BE, base excess; FiO_2_, oxygen inspiratory fraction; PEEP, positive airway expiratory pressure; Pplat, plateau inspiratory pressure; V_T_, tidal volume. Data expressed as mean ± standard deviation

**Table 3:** Hemodynamic data before and after MRS

	24 h preceding MRS	First 24 h after MRS
MAP (mmHg)	82 ± 9	86 ± 11
Arterial Lactate (mg/dL)	16 ± 10	14 ± 10
ScvO_2 _(%)	80 ± 8	85 ± 10
Fluid Balance (ml)	4,050 ± 2,724	2,538 ± 2,270

MAP, mean arterial pressure; lactate, arterial lactate; ScvO_2_, central venous saturation. Fluid balance, net fluid balance. Data expressed as mean ± standard deviation.

Eighteen patients were excluded because of the following reasons: hemodynamic instability judged to represent high risk for transportation (six patients), absence of informed consent (three patients), barotrauma detected before transportation (one case due to thoracic trauma and one case due to mechanical ventilation - before MRS), morbid obesity in no condition for transportation (two patients), more than 72 h of ARDS onset (two patients), do-not-resuscitate orders (one patient), age above 80 years (one patient) and pregnancy (one patient).

Fifty-one severe ARDS patients were included and followed, of whom 84% had primary ARDS. Community-acquired and nosocomial pneumonia represented 57% of the causes. Acute Physiology and Chronic Health Evaluation II (APACHE II) score was 20 ± 6, and Day-1 Sequential Organ Failure Assessment (SOFA) score was 10 ± 3. Non-pulmonary organ failure (NPOF) was 2 (IQR: 1 to 4) and 57% of patients required continuous renal replacement therapy. Seventy-one percent of the patients fulfilled sepsis criteria (89% of whom fulfilled septic shock criteria). Eighty-two percent of the patients required norepinephrine.

### Opening pressures

During MRS, Maximum PEEP was 45 (IQR: 43 to 45) cmH_2_O and Maximum Plateau Pressure was 60 (IQR: 58 to 60) cmH_2_O. In 13 cases, opening pressures (that is, the plateau-pressures associated with massive recruitment, during visual assessment) were lower than 60 cmH_2_O: 45 cmH_2_O (one patient), 50 cmH_2_O (five patients), and 55 cmH_2_O (seven patients).

In a preliminary, quantitative, CT analysis performed in the first 12 of our 51 patients, we observed 55% (IQR: 39 to 61) of non-aerated tissue at PEEP of 10 cmH_2_0 before MRS. The non-aerated tissue decreased progressively to 23% (IQR: 15 to 35) after reaching plateau-pressures of 40 cmH_2_O (at PEEP of 25 cmH_2_0), to 10% (IQR: 4 to 21%) after reaching plateau-pressures of 50 cmH_2_O (at PEEP of 35 cmH_2_0), and to 5% (IQR: 2 to 10) after reaching plateau-pressures of 60 cmH_2_O (at PEEP of 45 cmH_2_0). After PEEP titration (mean PEEP level of 23.7 ± 2.3 cmH20) the non-aerated tissue was kept at 7% (IQR: 3 to 13) [33].

### CT analysis and the size of the potentially recruitable lung

**Figure 2:**
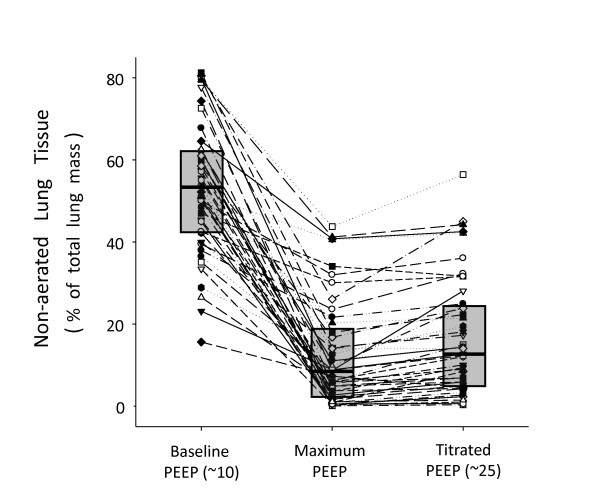
**Amount of non-aerated tissue observed along the protocol**. Numbers represent the percentage of non-aerated tissue (in relation to the total lung tissue) within the thick lung section (7 to 8 cm thick) from the carina to the diaphragm. Dashed lines represent the 45 individual cases and boxes represent the interquartile range, with a central line representing the median value at each moment.

**Figure 3:**
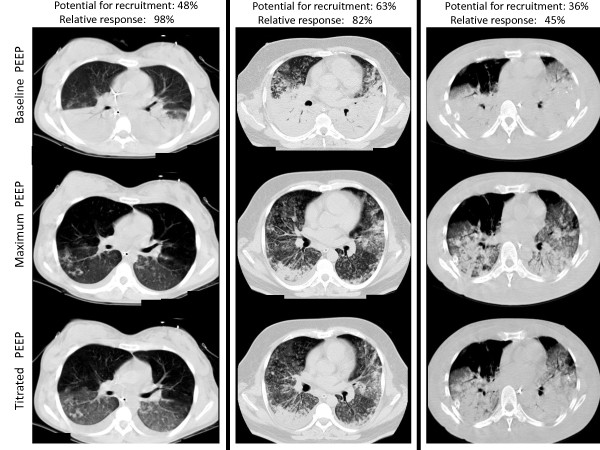
**Illustrative cases of MRS**. One with best relative-response (98% - left column), one with typical median response (82% - mid column), and another with the worst response (45% - right column). As can be noticed, the measurement of the potentially recruitable lung does not match this relative response (with values of 48%, 63% and 36%, respectively), since this last parameter is also strongly coupled with the amount of non-aerated tissue at baseline PEEP (49%, 78% and 80%, respectively).

Fifty-one patients completed the visual assessment of recruitment by CT, but only 45 patients had complete data for quantitative CT analysis. The amount of non-aerated tissue (percentage of lung mass) at minimum PEEP was 53.6% (IQR: 42.5 to 62.4), which decreased to 8.7% (IQR: 2.7 to 17.9) and 12.7% (IQR: 4.9 to 24.2) at maximum-PEEP and titrated-PEEP, respectively (Figure 2). Illustrative cases of MRS are depicted in Figure 3.

**Figure 4:**
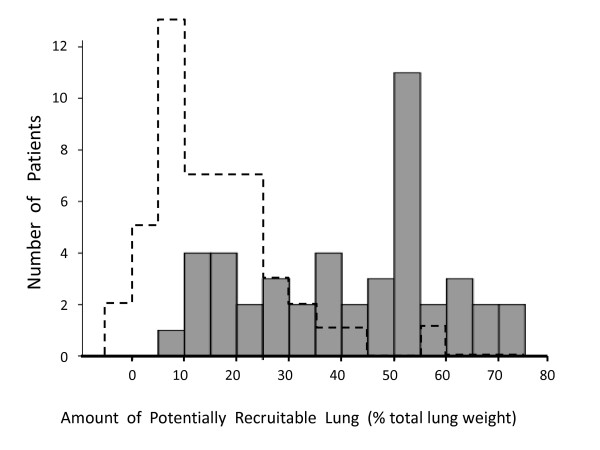
**Histogram for the amount of potentially recruitable lung observed in our sample of patients (N = 45)**. Dashed lines represent the equivalent histogram described by Gattinoni and cols. in a previous publication [29].

The histogram illustrating the size of the potentially recruitable lung in our sample is shown in Figure 4, with a median value of 45% (IQR: 25 to 53). Although there was correlation between the amount of non-aerated tissue at baseline and the potential for lung recruitment (*P *< 0.001, r^2 ^= 0.54), the relative response to MRS was not correlated with the initial amount of non-aerated tissue (*P *= 0.24, r^2 ^= 0.03).

### Gas exchange

**Figure 5:**
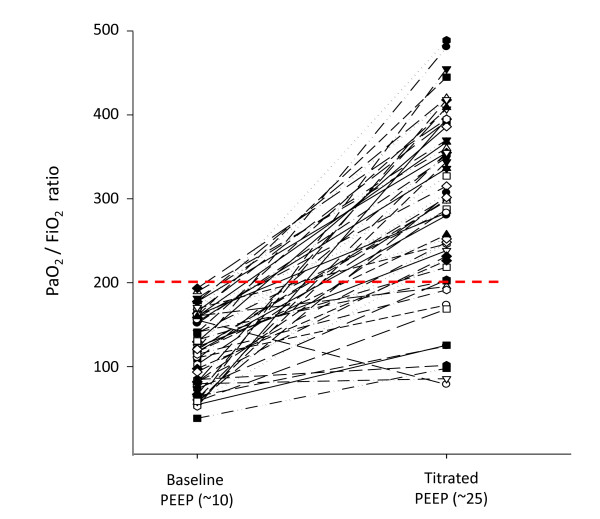
**Individual variations of PaO_2_/FIO_2 _ratio, before and after MRS**. The red dashed line represents the threshold of 200. Whereas the baseline blood gas was collected at 100% oxygen fraction, the second one, collected at the titrated-PEEP, was collected at an average FIO_2 _= 45% (± 17%).

**Figure 6:**
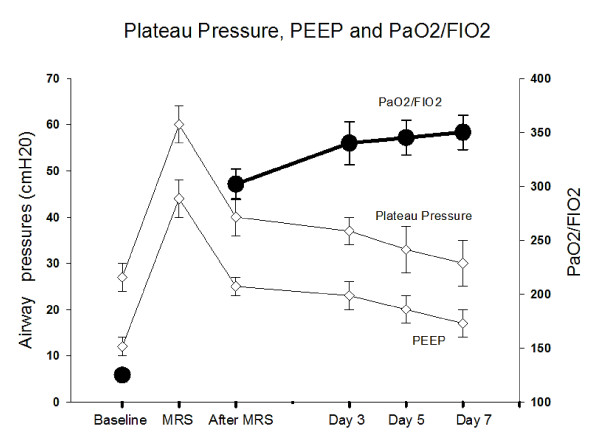
**Evolution of PaO_2_/FIO_2 _ratio, plateau pressures and PEEP levels throughout seven days**. Observe the relation among the variables before, during and throughout the seven days after MRS.

The response of the PaO_2_/FIO_2 _ratio to MRS is shown in Figure 5. Only seven patients did not reach a PaO_2_/FIO_2 _ratio > 200 after MRS. The seven-day evolution of PaO_2_/FIO_2 _is shown in Figure 6. The PaCO_2 _and arterial pH seven-day evolution is shown in Table 2.

### Ventilation parameters after MRS

After titration of PEEP, mean PEEP level was maintained at 24.6 ± 2.9 cmH_2_O and plateau-pressures at 40.0 ± 4.3 cmH_2_O. According to the patients' evolution, PEEP and plateau-pressure progressively decreased to 17.2 ± 3.7 and 29.7 ± 6.3 respectively on Day 7, while mean PaO_2_/FIO_2 _was kept above 300 (Figure 6).

### Evolution and outcome

Forty patients were successfully weaned, of whom 27 were extubated after a median duration of mechanical ventilation of 9 (IQR: 7 to 13) days. Thirteen patients were tracheostomized. Length of ICU-stay and hospital-stay was 15 (IQR: 12 to 33) and 30 (IQR: 21 to 63) days, respectively. ICU mortality was 28% (14/51) and hospital mortality was 33% (17/51).

Mortality in our sample was not associated with a larger potential for lung recruitment (*P *= 0.33), nor with a larger mass of non-aerated tissue at baseline (*P *= 0.81). The APACHE score did not differ between survivors and non-survivors (19 ± 8 versus 21 ± 5, respectively; *P *= 0.29). From Day 1 to Day 7, the SOFA score decreased significantly in the survival group (from 9 ± 3 to 4 ± 4; *P *< 0.001), but not in the non-survival group (from 10 ± 3 to 10 ± 4). Non-survivors were older than survivors: 61 ± 14 versus 47 ± 16 years (*P *= 0.009).

During Day-1, the titrated PEEP and tidal-volumes were similar between survivors and non-survivors (24.6 ± 2.3 versus 24.8 ± 4.1 cmH_2_0; *P *= 0.89; and 6.9 ± 1.3 versus 6.8 ± 1.7 mL/Kg; *P *= 0.68), but Day-1 plateau-pressures and Day-1 driving-pressures were higher in non-survivors when compared to survivors (43 ± 6 versus 39 ± 3 cmH_2_0; *P *= 0.039; and 17.0 ± 3.2 versus 13.8 ± 2.6 cmH_2_0; *P *= 0.002).

The PaO_2_/FIO_2 _ratio after MRS was higher in survivors versus non-survivors (324 ± 96 versus 239 ± 95.8 mmHg; *P *= 0.015), but not the PaCO_2 _(53 ± 16 versus 62 ± 17 mmHg; *P *= 0.13).

Cumulative fluid balance from entry till before MRS, or from entry till 24 hours after MRS was not different between survivors and non-survivors (+2,314 ± 1,988 mL versus +1,945 ± 2,297 mL; *P *= 0.78; or 3,715 ± 2,863 versus 5,012 ± 2,244 mL; *P *= 0.09; respectively).

It is noteworthy that the multivariate logistic regression analysis revealed only two independent predictive factors for mortality: older age (*P *= 0.007) and higher inspiratory driving pressure (inspiratory delta pressure-control; *P *= 0.011). Other variables tested in the multivariate model, which did not show significance were: Day-1 PEEP (*P *= 0.16), Day-1 plateau-pressure (*P *= 0.17), Day-1 PaCO_2 _(*P *= 0.44), Day-1 FIO_2 _(*P *= 0.19), or Day-1 PaO_2_/FIO_2 _ratio (*P *= 0.31)

### Side effects and complications

There were no major complications secondary to transportation to the CT-room, or during MRS. Minor problems detected during transportation were: malfunctioning of infusion IV pumps with vasopressors (and consequent transitory hypotension), malfunctioning of transportation monitor, EKG electrodes and arterial pressure curves. One patient developed acute atrial fibrillation without hemodynamic instability, which was pharmacologically reversed. No barotrauma was detected during MRS in the CT-room, or immediately after transportation to the ICU. Transient decreases in arterial blood pressure occurred during MRS without need of interruption of the maneuver because of persistent hemodynamic instability. Pneumodiastinum was detected in two patients, but at more than 48 h after MRS. Both coincided with reduction in sedation and detection of patient-ventilator asynchrony by the investigators at bedside. After the detection of pneumomediastinum in the chest X-ray of these two patients, we only adjusted the patients' sedation, guaranteeing a better patient-ventilation synchrony. We did not change mechanical ventilation strategy and patients were kept following the pre-established protocol.

## Discussion

The main findings of this study can be summarized as follows. MRS was a safe strategy to reduce the amount of non-aerated tissue in our non-selected population of severe ARDS patients. In order to obtain and sustain expressive recruitment, opening pressures above 45 cmH_2_O uninterruptedly followed by high PEEP were necessary, according to individual titration. By using MRS as a referential maneuver, the median percentage of potentially recruitable lung was 45%, a considerable number when compared to previous studies [29], but a higher amount of non-aerated tissue at baseline did not predict a worse response. All CT-scans were analyzed after MRS and no signs of minor or major barotrauma were observed in any patient. No significant clinical complications associated with MRS were noted either.

Recently, Gattinoni and colleagues [29] found that ALI/ARDS patients with a higher mass of non-aerated tissue and a higher potentially recruitable lung had increased mortality. They also demonstrated that the potentially recruitable lung varied widely in their population with a mean value of 13 ± 11% (considering the total lung mass). We believe that the main reason for the differences in relation to our study was the design of the recruitment maneuver *per se*: in Gattinoni's study [29], plateau inspiratory pressure was limited to 45 cmH_2_O and the associated PEEP was 5 cmH_2_O. Besides, there may be important differences in the studied population, since the patients in Gattinoni' s study had a mean duration of invasive mechanical ventilation of 5 ± 6 days when they were submitted to their recruitment protocol, while all our patients had less than 72 hours of ARDS onset. As demonstrated by Borges and colleagues [22], the maintenance of appropriate PEEP during the recruitment phase, above closing pressures (commonly above 15 cmH_2_O), is an essential component of recruitment maneuver, enhancing the recruitment effects of high plateau inspiratory pressures. Thus, the use of a low PEEP by Gattinoni and colleagues (5 cmH_2_O) interposed during their "recruitment phase" probably counteracted the net efficacy of the maneuver, promoting cyclic de-recruitment during this critical phase. Additionally, previous studies and the present one suggest that a maximum recruiting pressure of 45 cmH_2_O was not enough to substantially re-aerate collapsed areas in severe ARDS [22,28,34]. In a preliminary CT analysis performed in 12 of our 51 patients, we observed the persistence of 22% of non-aerated tissue at airway pressures around 40 cmH_2_O (accompanied by appropriate PEEP), in patients who later achieved less than 5% of lung collapse after MRS [33].

Our CT analysis revealed a four-fold reduction in the amount of non-aerated tissue after MRS (Figure 2), which means that the size of potentially recruitable lung in severe ARDS may be much higher (3.5 times) than previously reported [29,35] (Figure 4). Thus, the assumption that just a minority of patients with ARDS can be recruited [29,36] must be reconsidered. It is worthy of note that every single patient presented some radiological response to MRS, and the relative response to the maneuver could not be predicted by the baseline CT evaluation. Although there was some correlation between the amount of non-aerated tissue at baseline and the potential for lung recruitment (*P *< 0.001, r^2 ^= 0.54), the relative response to MRS was not correlated with the initial amount of non-aerated tissue (*P *= 0.24, r^2 ^= 0.03). Whereas the former, significant correlation likely reflects some mathematical coupling, that is, sicker patients have more room to improve, the latter, non-significant, correlation implies an important message: the initial CT quantification, even when showing impressive amounts of collapse, cannot rule out the chances of near-complete collapse reversal after MRS.

This interesting observation may explain why we did not find a correlation between mortality and the potential for lung recruitment in our patients, as previously reported by Gattinoni and Caironi [29,35]. If many of our patients with the highest potential for lung recruitment had near-complete reversal of collapse, the deleterious effects of lung collapse might have been especially counteracted in this subgroup.

Although the present study was designed to be a prospective case-series testing the MRS as described by Borges and colleagues [22], the two protocols were not identical. Some slight differences may explain the apparent higher recruitability of their patients, although the exact numbers are not provided in their publication. First, this case series had more strict inclusion criteria, but a less strict exclusion one. Thus, the present study included sicker patients, better representing the daily activities of a general ICU center. Secondly, the recruitment strategy in Borges' study had one additional recruitment step (CPAP of 40 cmH_2_0 for 40 seconds), with a longer duration of the intensive recruitment protocol: the total duration of their maneuver was 20 minutes (five steps of high inspiratory pressures, alternated with five steps of 25 cmH_2_0 PEEP resting periods - each step with 2 minutes), as compared with a total duration of 8 minutes in the present study (four steps of progressive, high recruitment pressures, with 2 minutes at each step, and without resting periods in between).

The immediate response in terms of oxygenation revealed a large increment in PaO_2_/FIO_2 _(Figure 5), especially in the first few hours after MRS. The overall improvement in the PaO_2_/FIO_2 _ratio was higher than previously reported in recent trials [13-15,37] (Figure 6). As a counterpoint to some reports in the literature suggesting that the effects of a recruitment maneuver in ARDS are transitory [38,39], our study showed that if PEEP is carefully selected after recruitment, the PaO_2_/FIO_2 _ratio can be maintained at high levels (> 300) during the first few days (or weeks) of mechanical ventilation. This finding suggests the possibility of long term maintenance of an open lung status, throughout the course of invasive mechanical ventilation, at an acceptable clinical cost [22].

By following the CT guided strategy and trying to select the minimum PEEP to keep the lungs open, we were forced to apply high levels of plateau-inspiratory pressures to our patients (between 30 and 40 cmH_2_O), in spite of using relatively low-tidal volume ventilation (6 to 8 mL/kg). Such levels are higher than the ones recommended by some protective protocols [13,14,40], and are certainly a matter of concern. We believe, however, that some of the findings in the present study, as well as in the recent literature, provide compelling arguments to counterbalance such concern. First of all, we did not find an important correlation between mortality and plateau-pressures in our study. This finding is in alignment with the results of some recent trials about PEEP selection, in which higher-PEEP arms consistently presented higher levels of plateau-inspiratory pressure [23] (especially in the Lung Open Ventilation Strategy (LOVS) study [15]), and yet, they resulted in similar or better outcome. Thus, the concerns about tidal hyperinflation or of excessive stress/strain [41,42] have always to be counterbalanced by the possibility of minimization of lung collapse with possible reduction of tidal-recruitment [35,43,44]. Secondly, overall mortality or barotrauma incidence in this study was comparable to the results of the best protective strategies reported in the literature [12-15,23,40,45], despite the higher severity of disease in our case-series. And finally, we kept our focus on minimizing the delta inspiratory-pressures in our patients and, in fact, this variable was found to be the most important predictor of survival (besides age) in our population. In most patients, we could keep it below 15 cmH_2_O, a relatively safe value according to preliminary evidences [46,47].

In retrospect, (maybe we could do better in terms of lung protection), trying to minimize further the delta-inspiratory pressures and plateau-pressures after the MRS could have done better in terms of lung protection. Nevertheless, except for some few cases in which we could have accepted more permissive values of hypercapnia, or could have optimized CO2 removal [48], the main constraints found during the realization of this protocol were related to PaCO_2 _levels, which averaged 56 (± 16 mmHg) during the first 24 hours after mechanical ventilation (resulting in a pH around approximately 7.20), and also related to the need of high PEEP levels, sustained along days after the MRS. In practice, we did not reduce PEEP before the first 48 hours, otherwise we would have had to increase the FIO_2 _(to > 40%), a procedure that we tried to avoid by protocol design.

Recently, Terragni and colleagues [41] showed that ventilation with tidal volumes around 6 ml/kg and "protective" plateau pressure (less than 30 cmH_2_O) were not enough to prevent what the authors defined as "tidal hyperinflation" in ARDS patients, especially in those patients with large amounts of non-aerated tissue at the dependent lung zones. The authors' conclusion was that collapse was an unavoidable phenomenon during ARDS, promoting the heterogeneity of tidal ventilation and hyperinflation of the baby lung, despite low tidal-volume ventilation. The authors suggested that the only possible strategy to prevent harm was the further decrease of airway pressures, even at the expense of deterioration in blood gases. Since this strategy is unfeasible in many patients, especially in those with severe hypoxemia, our study suggests that an alternative strategy is conceivable. MRS was particularly effective in those patients presenting larger amounts of collapse at baseline. Although not specifically tested in this study, the subsequent reversal of collapse might promote a more homogeneous distribution of tidal ventilation, making use of the previously collapsed parenchyma to "share" the tidal volume, and possibly relieving nondependent lung hyperinflation described by Terragni and colleagues [41]. This hypothesis was already suggested by others [22,34,49]. It is worthy of mention that in a detailed CT analysis performed in the first 12 ARDS patients of our protocol, the overall amount of tidal hyperinflated tissue increased by only 1% (± 1%) after MRS, despite a large increase in PEEP from baseline (approximately 10 cmH_2_O) to the titrated PEEP (approximately 25 cmH_2_O) [33].

Recently, Talmor and colleagues [37] monitored esophageal pressure in ARDS patients and observed that even with PEEP levels around 18 cmH_2_O and plateau pressures above 30 cmH_2_O, transpulmonary inspiratory pressures were kept below reasonable levels (< 12 cmH_2_O). They also showed that in order to keep the lung open, with transpulmonary expiratory pressures above zero, PEEP had to be kept above 15 cmH_2_O. Similarly, many of our patients were ventilated with plateau pressures above 30 cmH_2_O and PEEP levels above 15 cmH_2_O during the first days of ARDS, but with driving pressures less than 15 cmH_2_O, as in Talmor's study [37].

### Limitations of the study

We could not scan the whole lung due to time constraints and radiation exposure, so we chose to limit CT images to a representative lung fraction, from the carina to the diaphragm. Although not representing the whole lung behavior, previous studies strongly suggested that such partial sampling is adequate for quantitative CT analysis [50].

In this case series we performed inspiratory/expiratory thoracic CT scans only in the first 12 patients. Having found an increment of inspiratory hyperdistension as small as 1% at a PEEP of 25 cmH20 after the MRS (when compared to a PEEP of 10 cmH_2_0 before MRS), we decided to limit the exposure to radiation, performing only expiratory images during MRS in the remaining 39 patients.

Few unstable, critically ill patients and some obese and complicated abdominal post-operative patients could not be transported to the CT facility and were not included in our study (18 out of 69). Thus, our non-selective sample of ARDS patients has to be put into perspective.

Although our ICU and hospital mortality was relatively low (28 and 33%, respectively), this case series was not designed to estimate long term mortality, limiting the conclusion to be drawn about the ultimate clinical benefit of MRS. The fact that the SOFA score did not decrease in the non-survival patients indicates that in this specific population a non-detectable factor perpetuated multiple organ failure and patients' death despite MRS.

By using echocardiography, Vieillard-Baron and colleagues [51] demonstrated the occurrence of acute corpulmonale in about 25% of their ARDS patients submitted to limited pressure ventilation (average PEEP 7 ± 3 cmH_2_O). In our study, we did not monitor the patients systematically with echocardiography. Nevertheless, even using much higher ventilatory pressures than in the above mentioned study, there was no clinical evidence of acute right ventricular failure, systemic refractory shock or interruption of MRS due to acute hemodynamic complications.

## Conclusion

In our severe ARDS patients with multiple organ failure, MRS was a safe strategy to reverse non-aerated lung parenchyma and hypoxemia in most of them, for extended periods of time. In this particular study, CT was instrumental to individualize the strategy, in order to achieve almost full recruitment and to titrate PEEP, keeping the lungs open. Our results indicate that the general principles of the MRS are a valid alternative to conventional ventilation, worth being tested in future randomized trials.

## Key messages

• The MRS was feasible and reversed hypoxemia and the non-aerated areas of the lungs for extended periods of time in 51 patients with early, severe ARDS, revealing a much larger lung recruitability than reported in previous studies.

• The response to MRS cannot be predicted *a priori *and has to be tested individually. The initial CT quantification, even when showing impressive amounts of collapse, cannot rule out the chances of near-complete collapse reversal.

• Hospital mortality in our case-series was associated with older age and higher driving inspiratory pressures, but not with higher plateau-pressures, nor with a larger potential for lung recruitment at baseline.

• During MRS, we did not observe barotrauma or significant clinical complications.

• MRS should be tested in a randomized, prospective, controlled, clinical trial.

## Abbreviations

APACHE II: Acute Physiology and Chronic Health Evaluation II; ARDS: Acute Respiratory Distress Syndrome; CT: computed tomography; EKG: electrocardiogram; FIO_2_: inspiratory oxygen fraction of oxygen; HU: Hounsfield Unit; IQR: interquartile range; MAP: mean arterial pressure; MRS: Maximum Recruitment Strategy; NPOF: non-pulmonary organ failure; PEEP: positive end expiratory pressure; P_closing_: minimum expiratory pressure to sustain recruitment; P_opening_: minimum inspiratory pressure to achieve recruitment; PxV curve: pressure × volume curve; ROI: region of interest; ScvO_2_: central venous saturation; SD: standard deviation; SOFA: Sequential Organ Failure Assessment; SpO_2_: pulse oxygen saturation; VILI: ventilator induced lung injury; VT: tidal volume.

## Competing interests

The authors declare that they have no competing interests.

## Authors' contributions

GFJM, CSVB, FS, REC, MF, RA and RHP participated in transportation and care of the patients included during the protocol. GFJM and DCBS performed quantitative CT analysis. GFJM, JBB, MBPA and CSVB participated in the design and coordination of the protocol and in the drafting of the manuscript. All authors read and approved the final manuscript.
